# Helping Others, Warming Yourself: Altruistic Behaviors Increase Warmth Feelings of the Ambient Environment

**DOI:** 10.3389/fpsyg.2016.01349

**Published:** 2016-09-05

**Authors:** Tian-Yi Hu, Jingyu Li, Huiyuan Jia, Xiaofei Xie

**Affiliations:** ^1^School of Psychological and Cognitive Sciences and Beijing Key Laboratory of Behavior and Mental Health, Peking UniversityBeijing, China; ^2^College of Education, Shanghai Normal UniversityShanghai, China; ^3^School of Business Administration, Capital University of Economics and BusinessBeijing, China

**Keywords:** altruistic behavior, altruistic performer, crisis, social distance, warmth perception

## Abstract

Altruistic behaviors typically improve the welfare of the recipient at the cost of the performer’s resources and energy. Do altruistic performers obtain any positive internal reward from altruistic behaviors? We conducted six experiments to explore whether altruistic behaviors could increase performer’s warmth perception of the ambient environment. The first three studies focused on crisis situations. A retrospective field study (Study 1, with Hurricane Sandy) and two laboratory studies (Studies 2a and 2b, with an earthquake scenario) found that people who helped others felt warmer of the ambient environment than people who did not. We extended to daily life situations and found that participants who performed helping behaviors in laboratory (either voluntarily in Study 3a or randomly assigned to in Study 3b) and passers-by who donated to a charity (Study 4) reported warmer perception of the ambient environment than those who did not. These findings suggested an immediate internal reward of altruism.

## Introduction

One who watereth will himself be watered.

Proverbs 11:25

When we talk about altruistic behaviors, we often talk about sacrifice and the potential costs and risks associated with it. At the individual level, altruism is often non-economic and even maladaptive to survival because the altruistic performer needs to share his/her own resources and energy with others without receiving explicit returns (Batson, 1991; Myers, 1993; de Waal, 2008). When disasters signal a shortage of resources, it seems unwise to behave altruistically. However, at many times, we quote the saying *the roses in her hand, the flavor in mine* to encourage more altruistic behaviors. Is this quote simply a fortune cookie comment? Could those who behave altruistically literally experience the “flavor” or other positive physical feelings, as implied in this quote? In the current research, we explored the effects of altruistic behaviors on individuals’ physical feelings, focusing in particular on warmth. Warmth is a fundamental need to humans and other primates (e.g., Harlow, 1958; IJzerman et al., 2015a). Moreover, warmth perception of the ambient environment is a typical variable that links the psychological and physical worlds. Specifically, we found that altruistic behaviors could lead the altruistic performer to increase his/her warmth perception of the ambient environment.

### The Functional Adaptabilities of Altruistic Behaviors

Researchers have tried to recognize the functional adaptabilities of altruistic behaviors despite the self-sacrificing nature of such behaviors. The kin selection theory (Hamilton, 1963; Hamilton and Axelrod, 1981) suggests that altruistic behaviors toward those with shared genes (i.e., offspring or relatives of the altruistic performer) could maximize genetic frequencies at the group level. The reciprocal motivation and social exchange theory suggests that an altruistic performer could expect future returns either directly from the recipient (Trivers, 1971) or indirectly from a third party (Nowak and Sigmund, 1998, 2000). The above two theories both focus on the long-term benefits for the altruistic performers (Gintis et al., 2003).

However, researchers have uncovered the phenomenon of altruistic punishment. Individuals chose to incur great cost to punish norm violators in a group (e.g., Henrich et al., 2006). Such choices were regarded as altruistic for they aimed at restoring fairness and protecting the group norm. Because there was usually no kin relative in the group and the research employed a one-shot game (Gintis, 2000; Boyd et al., 2003; Gintis et al., 2003; Henrich et al., 2006), this phenomenon could not be explained by the two previously mentioned theories. Moreover, a neuroscientific study on altruistic punishment found that effective punishment activated the dorsal striatum. Participants with stronger activation in this brain area were willing to perform more punishment (de Quervain et al., 2004). As the dorsal striatum is an important part of the reward system (Knutson et al., 2000; Delgado et al., 2003), the findings implied that altruistic punishment could trigger immediate positive experiences for altruistic performers. This finding was consistent with earlier notions that altruistic behaviors would promote the release of endogenous opioid peptides (Danielli, 1980), which contributed to the control of pain (Basbaum and Fields, 1984) and the modulation of human mood and feelings of well-being (Leknes and Tracey, 2008). Furthermore, these findings implied that we may focus on the internal rewarding system of human altruistic behaviors. Rather than uncover tangible returns as a result of altruistic behaviors, we would like to explore the potential positive effects of altruism on one’s psychological and physical experiences.

Researchers have found positive psychological consequences of altruistic behaviors. For example, prosocial spending including charity donations and gift giving were found to evoke happiness (Dunn et al., 2008). Researchers in this field used the term “warm glow” to indicate an internal sense of satisfaction in donors after donating money (Harbaugh, 1998). Altruistic behaviors also promoted self-efficacy in the elderly (Midlarsky and Kahana, 1994) and enhanced positive self-evaluations (Post, 2005). In the current study, we focused on the warmth perception of the ambient environment. We chose this variable for three reasons. First, warmth is a fundamental need of humans and other primates. This has been supported by earlier studies on development and attachment (e.g., Harlow, 1958) as well as by a recent model of thermoregulation (IJzerman et al., 2015a). Feeling of warmth could be a source of security (Harlow, 1958) and individuals could use feeling of warmth as an indicator of social resources (IJzerman et al., 2015a). Research on winter depression implied that lack of warmth could be a threat to mental health (e.g., Molin et al., 1996). If altruism were to lead to threats against other survival-related resources such as food, compensatory feelings of warmth could be a comfort for individuals who are facing crisis situations, which could be an advantage for survival. Second, it is implied from previous research that the reward of altruism might be complicated. Reward, for instance, might be related to an internal reward system rather than simply tangible resources. Warmth perception of the ambient environment is a variable that links the psychological and physical worlds. It describes individuals’ internal reflections of the surrounding world, which could be an important facet of positive feedback of altruism. Third, coldness is a typical threat in many crises, especially in natural environments. For example, extreme cold weather in winter could cause a large amount of deaths (e.g., Webb, 2014; Ward, 2015). Coldness is also one of the most important environmental factors in mountain sports accidents (Chamarro and Fernández-Castro, 2009). Compared with non-crisis situations, people are more likely to lack resources (e.g., warm food, warm clothes) in coping with coldness. Moreover, individuals may tend to amplify the threats of the crisis-situations. Such tendency was usually related to other psychological reactions toward disasters such as post-traumatic stress disorder (e.g., Heir et al., 2009). And the tendency could also be amplified in a social level (Kasperson et al., 1988). Under extreme circumstances, individuals can do little to change the objective situation. Regulating warmth feelings of the ambient environment is potentially helpful for individuals to feel positively about the situations. We hypothesized that altruistic behaviors would increase the feelings of warmth among altruistic performers.

### Altruistic Behaviors Promote Physical Warmth

Researchers have linked feelings of warmth to social behaviors and social cognitions. Recently, IJzerman et al. (2015a) proposed a social thermoregulation model. According to this model, thermoregulation is costly for a single individual; therefore, social interactions (e.g., bodily contact) are vital and economic for animals to maintain proper body temperatures. The model argues that the process of social thermoregulation has shaped high-order social cognition. Earlier theories on grounded cognition (Barsalou, 1999, 2008; Schubert, 2005) have suggested that abstract social cognitions are grounded in interactions with the physical world. Metaphoric models emphasize metaphoric mappings between social cognition and physical perception (Landau et al., 2010). These theories help explain the connections between the social and physical worlds in human beings.

An important line of research has focused on a bidirectional relationship between physical warmth and social warmth. On the one hand, a well-known study conducted by Williams and Bargh (2008) showed that a simple manipulation of physical warmth could lead to favorable interpersonal evaluations and behaviors. In addition, Kang et al. (2011) found that physical warmth increased trusting behavior. Such behaviors were regarded conceptually as interpersonal warmth in social life. On the other hand, other studies have yielded evidence that social-related concepts raised warmth perception. For example, research found that thinking about traits and objects that were positively related to communal concepts raised perceived warmth of the ambient environment (Szymkow et al., 2013; IJzerman et al., 2015b). Moreover, neuroscientific studies have also supported connection between social warmth and physical warmth. Kang et al. (2011) found insula activation when physical warmth increased trusting behavior. Inagaki and Eisenberger (2013)’s study also found that social-warmth and physical-warmth conditions overlapped on their activations in ventral striatum and middle insula. This overlap was specific in warmth-related positive feelings.

Furthermore, this social-physical link of warmth has been indirectly supported by the “cold” side of social life. Specifically, Zhong and Leonardelli (2008) found that recalling a past experience of social exclusion resulted in a lower estimate of ambient temperature. A social exclusion manipulation could lower skin temperature, and physical warmth experiences were more desirable and effective in comforting the participants experiencing feelings of loneliness (Bargh and Shalev, 2012; Ijzerman et al., 2012).

Through the consistently observed relationship between social behaviors (especially positive or prosocial behaviors that foster social relationships) and feelings of warmth, we expected a similar relationship between altruistic behaviors (as typical prosocial behaviors) and warmth perception. Apart from happiness, self-efficacy or some other psychological states, altruistic behaviors could also lead to some positive consequences that are more physical related. Specifically, we predicted that altruistic behaviors would lead to an increased warmth perception of the ambient environment.

### Perceived Social Distance as a Mediator

Apart from the research that confirmed a relationship between social warmth and physical warmth, many studies have identified the effects of social distance and physical distance on warmth feelings. For example, IJzerman and Semin (2010) found that a closer perception of social distance (either induced by physical distance or by similarity manipulation) resulted in a higher estimate of ambient temperature. The effects could be reasoned that there is a similar overlapping of social distance and physical interpersonal distance (e.g., IJzerman and Semin, 2009), and that physical warmth is usually related to physical interpersonal distance.

In a recent model, social baseline theory was built on previous theories of attachment and other related findings (Beckes and Coan, 2011). The theory proposes that social relationship and social proximity is actually a baseline for human being because it is energy-saving and risk-reducing. Individuals are actually more activated when social relationships are threatened, and they have a tendency to return to “baseline.” Thus, individuals may incorporate relationship-oriented social behaviors to achieve social proximity. Because of the close link between social distance and warmth perception, social distance could be a proximal bridge between prosocial or relationship-oriented behaviors and warmth perception. Thus, we hypothesized that altruistic behaviors would increase the performers’ warmth perceptions of the ambient environment. Moreover, we predicted that this effect would be mediated by decreasing the perceived social distance between altruists and recipients.

### Overview of the Current Research

In the current research, we conducted six studies to test the hypothesis that altruistic behaviors would result in a warmer perception of ambient environment and to additionally test the effect of perceived social distance as a mediator. Study 1 was a retrospective field study conducted in the context of Hurricane Sandy, revealing the relationship between altruistic behaviors and perceived warmth in a crisis. Then, in Studies 2a and 2b, we created a crisis situation in the laboratory to replicate this effect and to test the mediation effect of perceived social distance. In Studies 3a and 3b, we extended such effect and the mediation effect of perceived social distance to daily life situations using laboratory experiments. In Study 4, we conducted a field study to replicate such an effect in a real donation activity. To note, all the studies in the current research were set in a comparatively cold environment. We did not explore how this effect would manifest in a much hotter environment.

## Study 1

Study 1 explored the effect of altruistic behaviors on feelings of warmth in a real crisis context. Hurricane Sandy was the most destructive hurricane of the 2012 Atlantic hurricane season, dramatically shocking 24 states in the United States with its ferocity dating back to 29th October, 2012. After the storm crashed ashore, the rain turned into blizzard conditions along the east coast of the United States. According to news reports, the highest snowfall accumulation was 36 inches (91 cm) (Kellogg, 2013). What was worse, due to the heavy snow, the heating and power systems were cut off for more than 10 days in some places in New York State. The extremely cold weather turned the recovery and reconstruction into chronic suffering. As a result, local residents were suffering the continuous stress of environmental threat in subsequent months. In this study, the sample included participants who were local residents at the time of the crisis and who experienced this crisis first-hand. We hypothesized that participants who recalled an altruistic experience (vs. those who recalled a non-altruistic experience) in the crisis would then have a memory of a warmer ambient environment.

### Method

#### Participants

G^∗^Power 3.1 was used to compute *a priori* power analyses in the study. According to Cohen’s (1992) suggestion, a power of 0.8 and an effect size that was slightly above moderate level (effect size *d* = 0.6) were used as the input data. The expected effect size was also consistent with previous research on similar topics (e.g., IJzerman and Semin, 2009, 2010). This resulted in an expected sample size of 72. Seventy-nine local residents from the disaster areas (New Jersey State and New York State) (33 males; *M_a_*_ge_ = 29.65 years, *SD* = 9.41) were recruited on Amazon’s Mechanical Turk website (MTurk^1^). They were asked to complete an online survey named “Life after Sandy Hurricane” and were each rewarded a $5 Amazon gift-card. The purpose of the survey was described as for exploring how Hurricane Sandy and the following extreme cold weather influence people’s daily life. The survey was launched within 3 months after Hurricane Sandy hit the United States while residents in the disaster areas were still suffering through the chronic recovery and reconstruction. Participants were randomly assigned to the altruistic or non-altruistic group. There were 42 participants in the altruistic group and 37 participants in the non-altruistic group.

#### Materials and Procedure

Participants were first informed that the survey aimed to explore how Hurricane Sandy and the subsequent extreme weather conditions influenced the local residents. Next, participants were asked to recall an experience during or after Hurricane Sandy and to write it down in detail (using at least 50 words). In the altruistic group, participants were asked to “recall an experience in which he/she did something mainly taking other people’s benefits into consideration (e.g., giving food to a homeless person on the street, offering someone a free-ride, volunteer work, etc.).” In the non-altruistic group, they were asked to “recall an experience in which he/she did something mainly taking his/her own benefits into consideration (e.g., rushing to the supermarket for necessities without thinking about how others may need the goods, refusing to offer a free-ride, ignoring neighbor’s asking for help, etc.).” Afterward, participants were asked to recall their instant feelings of the ambient environment after the experience along a 7-point scale (1 = *extremely cold*, 7 = *extremely warm*). As control variables, the participants were also asked to report their feelings of warmth in the current environment along a 7-point scale (1 = *extremely cold*, 7 = *extremely warm*) in addition to the estimated temperature (in degrees Fahrenheit) of the current environment.

### Results and Discussion

The participants recalled warmer ambient environment after the recalled altruistic experience (*M* = 4.55, *SD* = 1.19) than after the recalled non-altruistic experience (*M* = 3.35, *SD* = 1.06), *t*(77) = 4.68, *p* < 0.001, *d* = 1.06. The effect remained significant when we included as covariates the warmth feelings and the estimated temperature of the current environment during the survey, *F*(1,74) = 19.93, *p* < 0.001, η^2^ = 0.21. Moreover, the warmth feelings or the estimated temperature of the current environment during the survey did not show significant group differences [warmth feelings: *M*_altruistic_ = 4.40, *SD* = 1.08, *M*_non-altruistic_ = 3.97, *SD* = 1.44, *t*(77) = 1.52, *p* = 0.134; environmental temperature: *M*_altruistic_ = 69.55, *SD* = 5.99, *M*_non-altruistic_ = 67.39, *SD* = 10.07, *t*(76) = 1.17, *p* = 0.246].

In summary, Study 1 primarily demonstrated the effect of altruistic behavior on warmth feelings in a retrospective real-world crisis. It is worth noting that the results for warmth feelings of the current environment did not reach significance, although in the same direction as for the remembered environment. This seemed not consistent with previous research when recalling a past experience of social exclusion affected current feelings of warmth (e.g., Zhong and Leonardelli, 2008). This was possibly due to individuals’ separation of the current feelings and remembered feelings. In addition, the study was less controlled due to the nature of the real crisis scenario. Thus, well-controlled experiments were conducted in the laboratory by measuring and manipulating altruistic behaviors, and warmth feelings were collected instantly after the behaviors.

## Study 2a

Study 2a was designed to replicate the effect of altruistic behavior on warmth perception of the ambient environment in crisis context with three improvements. First, we created an earthquake scene in the laboratory. Participants were asked to imagine that they were stuck in an earthquake scene in which a stranger was requesting help. Second, the temperature of this laboratory-based earthquake scene was kept stable at 15°C. Third, altruistic willingness was directly measured in this study so that we could observe the relationship between altruistic willingness and warmth feelings. To match the settings of the scenario, participants took the experiment in groups of 4 and there was no former acquaintance within each group. Such design could increase a sense of reality and offer a closer simulation of the scenario. It could also be more natural to induce helping behaviors in such conditions. We expected that the participants with higher altruistic willingness would perceive higher levels of warmth of the ambient environment.

### Method

#### Participants

Because of the correlational nature of Study 2, a power of 0.8 and a medium level of *r* (*r* = 0.3) were used to compute the expected sample size (Cohen, 1992). This resulted in an expected sample size of 67. Study 2a took place in a university in Beijing, China. We recruited 69 college students (24 males; *M_a_*_ge_ = 22.31 years, *SD* = 2.97) from the campus online forum. All participants read the informed consent document and agreed to participate in the experiment for a payment of 10 RMB (approximately $1.5).

#### Materials and Procedure

As mentioned before, the procedure was implemented in groups of four participants with no former acquaintances. They were first guided into a preparation room where they read a brief introduction of the experiment and then led to the experiment room. The experiment room was set as a scene after an earthquake, with a constant temperature of 15°C and all lights turned off. Participants were asked to sit in a circle on the floor during the whole experiment. They each were approximately 40–50 cm apart from one another.

After ensuring that each of the four participants was ready, the laptops started synchronously to play a video containing standardized instructions leading participants through the whole experiment. Participants were asked not to interact with other participants. The first part of the video was a 40-s video clip of a real earthquake. Participants were instructed to imagine that they had just experienced the earthquake and were currently stuck in a room under a collapsed building with several tourists.

In the second part, the video displayed a questionnaire with several slides, and participants were asked to write down their responses on the paper. First, they were required to report how was their perception of the ambient environment along an 11-point scale (0 = *extremely cold*, 10 = *extremely warm*). This was served as a pre-altruism measure of warmth perception. Perceived severity and uncertainty of the situation were also measured as control variables. Second, participants were told that “one of the tourists did not have food and had requested help.” Participants were asked to “indicate (with a whole number between 0 and 100) the percentage of food they would like to share with the tourist.” The willingness-to-help was regarded as participants’ subjective willingness of altruistic behavior. Additionally, warmth perception of the ambient environment was again measured as a post-altruism measure. Finally, participants were debriefed and then paid for their participation.

### Results and Discussion

To control for individual differences in feelings of warmth before altruistic behaviors, we subtracted the pre-altruism measure from the post-altruism measure of warmth perception and used this result in analyses. Regression analysis showed that willingness-to-help was positively related to change in warmth perception of ambient environment, *F*(1,67) = 4.39, *p* = 0.040, β = 0.25. This relationship remained significant when perceived severity and uncertainty of the scenario were controlled in the first step of the regression, *F*(1,65) = 4.46, *p* = 0.039, β = 0.26.

Study 2a replicated an association between altruism and warmth perception of ambient environment, demonstrating that people with a greater subjective altruistic willingness would experience warmer feelings. However, limitations remained in the measurement of altruism. First, neither Study 1 (recalled altruistic behavior) nor Study 2a (altruistic willingness) measured actual altruistic behavior. Second, the results of Study 2a were correlational, so a causal relationship between altruism and warmth feeling was not confirmed. Third, it remained unclear why the effect of altruism on perceived physical warmth emerged.

## Study 2b

Study 2b aimed at confirming the causal relationship between altruism and warmth perception in a crisis situation. Participants were randomly manipulated to exhibit an altruistic or a non-altruistic behavior in an experiment. We also explored a possible mediator of the relationship, hypothesizing that participants in the altruistic group (vs. the non-altruistic group) would report a warmer perception of the ambient environment because they felt a closer social distance to the help seeker. Moreover, we measured participants’ prosocial traits to control for the possible confounding effects of individual differences.

### Method

#### Participants

The computation of the sample size was identical to that of Study 1, resulting in a sample size of 72. Study 2b took place in a university in Beijing, China. Eighty-five university students were recruited from the campus online forum and randomly assigned to one of the two conditions (altruistic vs. non-altruistic). Five participants were excluded from the final data analysis because they responded to less than two-thirds of the assessment items. The final sample (28 males; *M_a_*_ge_ = 22.41 years, *SD* = 3.04) comprised 36 participants in the altruistic condition and 44 participants in the no-altruistic condition. Participants each received a payment of 10 yuan RMB (approximately $1.5).

#### Materials and Procedure

The settings and procedure of Study 2b were almost identical to those of Study 2a except for several important changes. First, although participants still participated in groups of four, one of the four participants in each group was actually a confederate.

Second, in addition to reading a brief introduction of the experiment in the preparation room, each participant was given a bag. They were told not to open and examine the bag until the instructions told them to do so in the experiment. The three bags for the real participants each contained ten small packs of bread (20 g each). There was no food in the confederate’s bag.

Third, the participants were guided to the experiment room, which had the same settings as in Study 2a. But after watching the first part of the video, the participants were instructed to open the bag and count the bread packs. Because participants were sitting close to each other (∼40–50 cm away from each other), they could easily notice that the confederate had nothing in the bag.

Fourth, participants were then instructed to report (1) the pre-altruism measure of warmth perception of the ambient environment and (2) control variables. Apart from the perceived severity and uncertainty of the situation, negative emotions (i.e., sadness, fear, desperation, and stress; α = 0.85) were also included as control variables.

Fifth, after the above measures were taken, participants were told that the person with no food in bag had requested help. Moreover, participants were manipulated into two groups by reading the following words: ‘*gathering everyone’s power and sharing resources with the group were the best choice in crisis’* (altruistic group) or ‘*saving one’s own resources and maximizing one’s own benefits were the best choice*’ (non-altruistic group). Each participant was asked to decide how many bread packs he/she would like to give to the help-seeker and to take the corresponding number of packs out of the bag. Participants were also instructed to write down the number on the answer sheet. This was used as the manipulation check of altruistic behavior.

Sixth, participants were asked to report perceived psychological distance with the other tourists in the situation on a 7-point scale (1 = *close*, 7 = *distant*) and this measure of social distance was used as the proposed mediator. The post-altruism measure of warmth perception was measured identically to Study 2a. Finally, participants completed the Social Value Orientation Questionnaire as a measure of prosocial traits after returning to the preparation room.

#### Prosocial Trait

We used the Social Value Orientation Questionnaire (Van Lange et al., 1997) to measure prosocial traits. This questionnaire contains nine multiple-choice situations. In each situation, a participant needed to choose one of three options to decide the outcomes for himself/herself and for another player. The three options in each situation corresponded to three social value orientations: competitive (seeking a larger difference between one’s own and other’s outcomes), individualistic (seeking a larger outcome for oneself), and prosocial (seeking a larger joint outcome). If the choices of six or more out of the nine situations were consistent with one of these social value orientations, participants were classified accordingly. Otherwise, the participant was designated as unclassified.

### Results and Discussion

The manipulation check of altruistic behavior was successful. Participants in the altruistic condition shared more packs of bread (*M* = 3.11, *SD* = 0.98) with the help-seeker than those in the non-altruistic condition (*M* = 2.55, *SD* = 1.13), *t*(78) = 2.36, *p* = 0.021, *d* = 0.53.

In terms of change in warmth perception of the ambient environment, the result was consistent with Studies 1 and 2a. Participants in the altruistic group reported more increase in warmth feelings of the ambient environment (*M* = 0.44, *SD* = 2.34) than those in the non-altruistic group (*M* = -0.70, *SD* = 2.53), *t*(78) = 2.09, *p* = 0.040, *d* = 0.47. The effect remained significant when negative emotions, perceived severity and uncertainty of the scenario were controlled as covariates in the analysis, *F*(1,74) = 4.33, *p* = 0.041, η^2^ = 0.06.^2^

A Chi-square test showed that there was a tendency that the distributions of the four social value orientations (competitive, individualistic, prosocial, and unclassified) in the two groups were not balance [χ^2^(3) = 6.43, *p* = 0.092]. We also tested whether social value orientations affect change in warmth perception. Because social value orientation was a categorical variable, we run a 2 (experimental manipulation: altruistic vs. non-altruistic) × 4 (social value orientation: competitive vs. individualistic vs. prosocial vs. unclassified) ANOVA. Because there was no significant interactions between the experimental manipulation and the social value orientation (*p* = 0.524), a custom model was run for analyzing only the main effects. Results showed that the effect of experimental manipulation remained marginally significant, *F*(1,75) = 3.39, *p* = 0.070, η^2^ = 0.04. And social value orientation did not significantly affect change in warmth perception, *F*(3,75) = 1.50, *p* = 0.232, η^2^ = 0.06.

Next, we examined whether perceived social distance mediated these effects. Participants in the altruistic group reported a significantly shorter perceived distance (*M* = 2.53, *SD* = 1.00) than those in the non-altruistic group (*M* = 3.16, *SD* = 1.46), *t*(78) = 2.20, *p* = 0.031, *d* = 0.50. Regression analysis showed that the group variable (the altruistic group coded as “1” and the non-altruistic group coded as “0”) was a significant predictor of warmth perception (β = 0.23, *p* = 0.040). However, the strength of this relationship became non-significant (β = 0.18, *p* = 0.105) when perceived social distance was included in the analysis. Moreover, perceived social distance had a tendency to be negatively related to warmth perception (β = -0.19, *p* = 0.091). The above results imply a mediation effect of perceived social distance. To further confirm the mediation effect, a 5000-sample bootstrapping analysis was conducted. Results showed that the 95% bias-corrected confidence interval for the indirect effect was [0.03, 0.61], suggesting a significant indirect effect (MacKinnon et al., 2007). Therefore, the results of Study 2b confirmed that engaging in altruistic behaviors can increase the perceived warmth of the ambient environment while also suggesting that reduced social distance could be an internal process.

So, the first three studies focused on the relationship between altruism (recalled altruistic behavior, altruistic willingness, and manipulated altruistic behavior) and perceived warmth of the ambient environment. The scenarios used in these studies were all crisis situations with low environmental temperatures. The temperature setting was important because a cold environment could be an extreme threat to survival during a crisis. Thus, regulating warmth perception of the ambient environment would be of great significance.

In the following three studies, we aimed to extend these findings to non-crisis situations with cold environments. This was mainly for two reasons. First, although we explored altruistic behaviors with either real or laboratory-based crisis situations, the manipulation and observation of altruistic behaviors suffered from some limitations. We could obtain only retrospective data from real crisis, and the manipulation of altruistic behaviors was direct. Second, participants in Studies 2a and 2b took the experiments in groups, which could result in interdependence of data. Although we did not find significant influence of group in Study 2b, following studies are aimed to avoid this problem by changing the experimental design. Third, the situations used in the previous studies were crisis-related, and we would like to confirm the relationship in less-threatening daily situations.

## Study 3a

Study 3a was designed to examine the association between altruistic behaviors and perceived warmth of the ambient environment in our daily life. We made two improvements in this study. First, we adopted temperature estimation as an additional index of warmth perception. Second, body temperature was measured as a control variable. We predicted that participants who chose to behave altruistically would report feeling warmer in the environment than those who chose not to help. In this study, we also predicted a mediation effect of perceived social distance.

### Method

#### Participants

The computation of the sample size was identical to that of Study 1, resulting in a sample size of 72. Study 3a took place in a university in Beijing, China and participants were college students recruited from the campus online forum. The final sample contained 64 participants (33 males; *M_a_*_ge_ = 22.66 years, *SD* = 2.47). Thirty-two participants who engaged in the altruistic task were labeled the altruistic group and the rest were labeled the non-altruistic group.

#### Materials and Procedure

The temperature of the experiment room was maintained constantly at 15°C. Seventy-one college students were recruited and completed a 10-min irrelevant decision-making questionnaire for a reward of 10 yuan RMB (approximately $1.5). After participants were paid, they were invited to participate in an additional activity organized by the Student Union of the Department of Psychology, but for no extra reward. Seven participants refused to participate and were excluded from the final analysis. Thus, the final sample contained 64 participants.

The additional activity involved two tasks for children from low-income migrant workers’ families. These children are usually regarded as a disadvantaged group in China. The first task was time-consuming and required considerable attention. Participants needed approximately 10 min to read and revise some educational materials for these children. The second task required participants to just complete a 1-min questionnaire about their understandings on these children. In this questionnaire, a 1-item measure of perceived social distance toward these children was presented along a 7-point scale (1 = *extremely close*, 7 = *extremely distant*) with some filling items. Participants could decide whether to participate in both tasks or in the second task only. Based on their decisions, participants were labeled as altruistic or non-altruistic.

Lastly, participants were asked to finish another small survey entitled “A survey on the Laboratory Environment.” In the survey, participants were required to report their perception of the warmth of the experiment room on an 11-point scale (0 = *extremely cold*, 10 = *extremely warm)*. They were also asked to estimate the room temperature in degrees Celsius. As a control, we measured the body temperature of the participants using a non-contact infrared thermometer. Finally, participants were debriefed and thanked for their participation.

### Results and Discussion

As expected, participants in the altruistic group felt the room was warmer (*M* = 6.06, *SD* = 2.18) than those in the non-altruistic group (*M* = 4.53, *SD* = 2.09), *t*(62) = 2.86, *p* = 0.006, *d* = 0.72. This effect remained significant when body temperature was included as a covariate, *F*(1,61) = 9.31, *p* = 0.003, η^2^ = 0.13. Participants in the altruistic group also reported higher estimates of the room temperature (*M* = 16.91, *SD* = 5.21) than those in the non-altruistic group (*M* = 12.58, *SD* = 4.48), *t*(62) = 3.57, *p* = 0.001, *d* = 0.89. This effect also remained significant when body temperature was included as a covariate, *F*(1,61) = 12.34, *p* = 0.001, η^2^ = 0.17. Moreover, there was a significantly positive correlation between feeling of warmth and estimate of the room temperature (*r* = 0.36, *p* = 0.002). However, body temperature did not correlate with feeling of warmth (*r* = -0.20, *p* = 0.120) or estimate of the room temperature (*r* = 0.05, *p* = 0.671). We then examined whether perceived social distance mediated the effects of group conditions on warmth perception. Participants in the altruistic group reported a significantly shorter distance (*M* = 3.66, *SD* = 1.54) than those in the non-altruistic group (*M* = 4.47, *SD* = 1.32), *t*(62) = 2.27, *p* = 0.027, *d* = 0.57. Regression analysis showed that the group variable (the altruistic group coded as “1” and the non-altruistic group coded as “0”) was a significant predictor of warmth perception, β = 0.34, *p* = 0.006). This relationship weakened (β = 0.28, *p* = 0.024) when perceived social distance was included in the analysis. Moreover, perceived social distance had a tendency to be negatively related to warmth perception (β = -0.21, *p* = 0.087). The above results implied a mediation effect of perceived social distance. To further confirm the mediation effect, a 5000-sample bootstrapping analysis was conducted. Results showed that the 95% bias-corrected confidence interval for the indirect effect was [0.04, 0.89], suggesting a significant indirect effect (MacKinnon et al., 2007).

In conclusion, the effect of altruistic behavior on perceived warmth and the mediating effect of perceived social distance were verified in a daily life situation. In Study 3b, we imported a stricter control of altruistic behaviors to confirm the effect of altruistic behavior on warmth perception of the ambient environment.

## Study 3b

In Study 3b, we randomly assigned participants to an altruistic or a non-altruistic condition. Moreover, a no-task group was added as a control group to rule out the alternative explanation that non-altruistic behavior could lead to a decrease in warmth perception of the ambient environment. We hypothesized that participants in the altruistic group would report higher warmth perception than those in the non-altruistic group and the no-task group. We expected participants in the non-altruistic and the no-task groups to report equal feelings of warmth.

### Method

#### Participants

Because Study 3b was designed to be consisted of three groups, a power of 0.8 and an effect size f of 0.35 were used to compute the expected sample size, resulting in a sample size of 81. Study 3b took place in a university in Beijing, China. Eighty-three college students were recruited from the campus online forum. Eight of them did not finish the experiment and were thus excluded from the analysis. The final sample contained 75 participants (36 males; *M_a_*_ge_ = 22.2 years, *SD* = 7.42). Participants were randomly assigned to one of the three conditions, resulting in 25 participants in each group.

#### Materials and Procedure

Participants were led to the experiment room, the temperature of which was maintained constantly at 15°C. In the altruistic condition, participants were invited to take part in a charity activity called ‘The Love Crossing 4000 Kilometers’ to help the students from a remote town called Jimunai. This town was one of the poorest areas in China, located 4000 km from Beijing. The charity aimed to collect stationery for these students and offer them more opportunities to learn about the outside world. Each participant was invited to write a postcard for a particular student in Jimunai Secondary School to introduce an attraction in Beijing. Participants then were given a 10-RMB (approximately $1.5) cash note and were required to put the note in a charity box. Notably, all of the postcards were sent to the corresponding students, and the money was donated to the school in the name of participants after the experiment.

In the non-altruistic condition, participants were asked to read information about Beijing’s candidature for the 2022 Winter Olympic Games. Participants were told that the laboratory was helping to advocate the attractions in Beijing. They could choose one postcard and write down an introduction about an attraction in Beijing. In the no-task group, participants were assigned no additional task.

All the participants then were asked to complete the survey about the laboratory environment, which was identical to that used in Study 3a. In the survey, participants were required to report their warmth perception of the experiment room along an 11-point scale (0 = *extremely cold*, 10 = *extremely warm*). As a control, we measured the body temperature of the participants using a non-contact infrared thermometer. Finally, all the participants were debriefed, thanked, and each paid 10 RMB (approximately $1.5) for their participation.

To ensure the successful manipulation of altruism, we asked another 78 participants (36 males) to read the scenarios in Study 3b and to then indicate the extent of altruism (‘To what extent do you feel you could help others if you participate in this activity?’ 1 = *not at all*, 5 = *great deal*). Results showed that the participants in the altruistic condition reported more helping (*M* = 3.63, *SD* = 0.75) than those in the non-altruistic condition (*M* = 3.18, *SD* = 0.90), *t*(76) = 2.42, *p* = 0.018, *d* = 0.55. To exclude the possibility that the non-altruistic material would induce thoughts of competition, the participants also indicated perceived competition (‘To what extent do you feel you are competing with others?’ 1 = *not at all*, 5 = *great deal*). No significant difference was found between the participants in the two conditions, *p* = 0.976. In addition, participants were required to write down 5–10 words that the reading material reminded them. We asked two experimenters who did not know the intention of the study to classify the words. Results showed that participants who read the altruistic scenario wrote down more altruistic-related words (*M* = 1.06, *SD* = 0.17) than those who read the non-altruistic scenario (*M* = 0.27, *SD* = 0.04), *t*(76) = 9.63, *p* < 0.001, *d* = 6.56. Similar results were found for caring-related words (*M*_altruistic_ = 1.13, *SD* = 0.70, *M*_non-altruistic_ = 0.08, *SD* = 0.27, *t*(76) = 8.85, *p* < 0.001, *d* = 2.02.

### Results and Discussion

One-way ANOVA revealed that the warmth perception of the participants were significantly different across three conditions, *F*(2,72) = 4.77, *p* = 0.011, η^2^ = 0.12. As expected, Bonferroni *post hoc* analysis showed that participants in the altruistic group (*M* = 6.84, *SD* = 1.89) felt significantly warmer in the room than those in the non-altruistic group (*M* = 5.32, *SD* = 2.01) (95% CI of mean difference [0.19, 2.85], *p* = 0.019). Likewise, participants in the altruistic group felt significantly warmer than those in the no-task group (*M* = 5.48, *SD* = 1.83) (95% CI of mean difference [0.03, 2.69], *p* = 0.042). However, there was no significant difference in warmth perception between the non-altruistic and no-task groups (95% CI of mean difference [-1.17, 1.49], *p* = 1.00). Moreover, the effect of condition remained significant when the body temperature of participants was included as a covariate, *F*(2,71) = 4.55, *p* = 0.014, η^2^ = 0.11. And there was no significant correlation between feelings of warmth and body temperature of the participants, *r* = 0.08, *p* = 0.490.

With stronger causal inference in this instance, the results replicated the effect of altruism on the warmth perception of the ambient environment. By adding a no-task control group, this study showed that the effects on warmth perception was caused by an increase in warm feelings from behaving altruistically rather than a decrease from not behaving altruistically.

Studies 3a and 3b replicated in the laboratory the primary findings of Studies 2a and 2b with regard to daily life situations. Compared with crisis situations, daily situations were more naturally created. In Study 3a, altruistic behaviors were chosen voluntarily by participants whereas in Study 3b, participants were randomly led to believe that they did an altruistic behavior or a non-altruistic behavior. The results provided convergent evidence for the proposed effect that altruistic behaviors would increase the perception of ambient warmth. To note, Studies 3a and 3b were underpowered by 8 and 6 participants compared to the computed sample sizes, respectively. This was due to experimental constraints of unexpected loss of participants and was a limitation for both studies. To further confirm the relationship and to foster external validity, we conducted another study in a real life situation to explore how a common altruistic behavior, donation, affected warmth perception of the ambient environment.

## Study 4

Study 4 was a field experiment conducted to increase the external validity of previous work and confirm the effects of altruistic behavior on the perception of warmth of the ambient environment. The experiment was conducted with the help of the Students Association of Sunshine Volunteers at Peking University. An actual charity event was held on December 7, 2012, to collect donations for children with leukemia. A donation desk was placed on the sidewalk along the campus main street. We randomly solicited individuals to complete a short questionnaire after they passed the donation desk. We hypothesized that people who donated (the altruistic group) would feel warmer regarding the ambient environment than those who did not donate (the non-altruistic group).

### Method

#### Participants

Because Study 4 was a field study, a power of 0.8 and an effect size d of 0.5 (which was smaller than previous studies) were used to compute the expected sample size, resulting in a sample size of 102. A total of 108 people participated in this experiment (47 males; *M_a_*_ge_ = 22.27 years, *SD* = 5.50). Of these, 55 made a donation.

#### Materials and Procedure

Data were collected from 9:00 a.m. to 1:00 p.m. during the day. The environment temperature during the experiment ranged from -7°C to 0°C. Participants filled in a short questionnaire called “Perception of Weather Conditions in Beijing,” which included items assessing warm feelings about the environment along an 11-point scale (0 = *extremely cold*, 10 = *extremely warm)* and an estimation of the environment temperature in Celsius degrees. To control for hourly temperature variations, we paired a passer-by who did not donate within 2 min after a donor completed the questionnaire.

### Results and Discussion

Consistent with our prediction, results showed that the passers-by who made a donation (altruists) perceived the ambient environment as warmer (*M* = 3.07, *SD* = 2.01) than those who did not (*M* = 2.40, *SD* = 1.86). The difference was marginally significant, *t*(106) = 1.81, *p* = 0.073, *d* = 0.35. The donors also reported significantly higher temperature estimations (*M* = -0.51°C, *SD* = 3.40) compared to the non-donors (*M* = -2.34°C, *SD* = 3.33), *t*(106) = 2.83, *p* = .006, *d* = 0.54).

## A Meta-Analysis

So far, we have consistently revealed the effects of altruistic behavior on physical warmth. For further verification, we conducted a meta-analysis to test the statistical replication of the experiments. The present experiments provided a good condition for such an analysis for two reasons. First, the altruistic behaviors varied in category, including sharing food with others, charitable helping, and monetary donations. Second, both college students in China and residents in America were included in the experiments, offering a significant diversity of participant populations.

Because Study 2a did not involve grouping participants, data from all other five studies were included. We used warmth perception of the ambient environment as the main dependent variable. Using Comprehensive Meta Analysis software, we entered the means, standard deviations, and sample sizes of the altruistic group and the non-altruistic group to calculate effect sizes. As shown in **Table 1**, the combined *z*-value was 5.94 (*p* < 0.001), which confirmed that altruistic behaviors increased physical warmth. Moreover, the heterogeneity test showed that the *q*-value was 6.24 (*p* = 0.182), indicating that the effects did not differ significantly across the studies.

**Table 1:** Results of a mini meta-analysis of the six studies.

Study	Standard difference in means	Standard error	*Z*-value	*p*-value
Study 1	1.061	0.241	4.407	0.000
Study 2b	0.466	0.228	2.046	0.041
Study 3a	0.716	0.258	2.778	0.005
Study 3b	0.779	0.293	2.656	0.008
Study 4	0.346	0.194	1.783	0.075
Combined statistics	0.627	0.106	5.943	0.000


## General Discussion

Through six experiments, we found that altruistic performers were likely to feel warmer about the ambient environment than those who either refused to help or did not have a chance to help. Furthermore, results revealed that this effect was mediated by perceived social distance (Studies 2b and 3a). Moreover, this effect was confirmed in both crisis and ordinary situations amidst different experimental settings. Altruism is a complicated construct. It is usually directed by voluntary motivations and thus altruistic behaviors were just observed in Studies 2a, 3a, and 4. To confirm the causal relationship, recalled altruistic experiences as well as instant altruistic behaviors were manipulated in Studies 1, 2b, and 3b. Moreover, different instructions and cover stories were incorporated in Studies 2b and 3b. In summary, this design of series studies complemented for possible weaknesses in terms of constructing altruism and offered convergent findings.

### Immediate Self-Reward of Altruistic Behaviors

While improving the welfare of others, altruistic behaviors typically deplete the energy and resources of the altruistic performers. Earlier explanations of altruism focused on its long-term return, either through genetic propagation (Hamilton, 1963; Hamilton and Axelrod, 1981) or reciprocity (Trivers, 1971; Nowak and Sigmund, 1998, 2000). However, research on altruistic punishment observed the activation of the reward system of the dorsal striatum, signaling an instant satisfaction from altruistic behaviors (de Quervain et al., 2004). Results from the current research have implied a potential self-reward mechanism for altruistic behaviors.

We found an immediate rewarding effect on the perceived warmth of the ambient environment after performing altruistic behaviors. In six studies, warm feelings were measured immediately after the participants reported the willingness to help and after they exhibited actual altruistic behaviors. This suggested that the increased feeling of warmth surrounding the ambient environment was an immediate reward rather than a long-term return-benefit for altruistic behavior performers. Furthermore, this effect was found to be mediated by perceived social distance, introducing a potential psychological process in this immediate reward. As mentioned in the introduction, previous research also found that altruistic behavior could result in some immediate psychological reward, including happiness (Dunn et al., 2008), self-efficacy (Midlarsky and Kahana, 1994), and positive self-evaluation (Post, 2005). Moreover, Dawans et al. (2012) found that exposure to acute social stress could increase prosocial responses, which implied an immediate protective function of altruism in coping with acute stress. In the current research, the immediate reward was found to extend to concrete physical feelings through a psychological process.

We proposed three characteristics of the immediate reward system of altruism that were different from those of its long-term benefits. First, an immediate reward is a much more certain and spontaneous effect compared with long-term benefits. An altruistic performer may end up with no real benefits in the long run but could always obtain positive feedbacks through the immediate reward system. Second, the immediate reward could operate on the psychological or even perceptual level. Compared with the external benefits from genetic propagation and reciprocity, the internal immediate reward could serve as a direct incentive to engage in altruistic behaviors. Third, receiving an immediate reward could be a self-feedback process. For long-term benefits, altruistic performers usually need to rely on the behaviors or the survival possibilities of others. In contrast, altruistic behaviors could directly activate the performer’s psychological and physical processes on the individual level. To summarize, an immediate reward from altruism could be certain, internally perceived, and self-activated, compared to long-term benefits.

The immediate reward for altruistic performers could be very valuable. The observed results of altruistic behaviors in previous research included positive emotions and positive self-cognitions, all of which were found to be positively related with people’s subjective well-being (e.g., Bandura, 1986; Brunstein, 1993). A meta-analysis found that volunteers had lower risks of mortality (Jenkinson et al., 2013). In the current research, we observed a consistent and direct connection between physical warmth and altruism. Physical warmth may be an important source of psychological energy or at least a strong comfort for individuals who were exposed to a cold environment. Specifically, individuals could perceive the environment as more secure and predictable (IJzerman et al., 2015a), and thus gain more confidence in coping with the environment. Thus, if the immediate reward from altruism could be valuable for the altruistic performers, they can be trusted because the reward would be more direct and certain.

In all, it suggests that altruism can not only bring long-term benefits to the performers (as explained in kin selection and reciprocity accounts) but also evoke immediate positive feelings inside the performers (as revealed in the current research). These two systems offer a better understanding of the functional adaptabilities of altruistic behaviors. Traditional economic analysis of altruism is based on a cost/utility analysis of external factors such as money, time, and probabilities of genetic propagations. We proposed to add internal utility (psychological states such as warmth perception and emotion) in the discussion of altruism. Altruistic behaviors would be evolutionarily meaningful if the increase in the internal utility offsets the reductions in external utility. Thus, the framework of altruism would be more complete when we concern both long-term and immediate benefits, as well as both external and internal utilities.

### Lingering Fragrance Effect

A potential immediate self-reward system of altruistic behaviors has practical meaning in promoting people’s well-being and quality of life in both daily life and times of crisis. The effect found in the current research was a typical example of the interaction between an individual’s physical and psychological system. The saying *the roses in her hand, the flavor in mine* could reveal a real psychological activation effect, through which people could promote their personal physical states. Thus, we named this psychological activation effect as a lingering fragrance effect.

When facing a threat (e.g., hunger, pain, or cold), there are mainly two strategies for its removal. One is to cope with the threat immediately (e.g., put on a coat to remove coldness). The other strategy is to activate a psychological process to change the perception of the threat. As in the current research, altruistic behavior activated people’s psychological processes and changed their warmth feelings of a cold environment. During crisis, the adverse external conditions could hardly be changed because of insufficient support of food, water, and living conditions. However, individual cognitions and behaviors are pliant and controllable, offering possibilities to attenuate the threats in crisis. Hence, psychological activations or subjective adjustments could become a prospective coping strategy under such circumstances. Importantly, the experimental settings used to induce people’s real altruistic behaviors might be artificial in the current research. Moreover, we found consistent lingering fragrance effects in spite of this shortcoming. Thus we expect in real life that the lingering fragrance effect of altruistic behaviors should be amplified to benefit individuals in a more extensive manner.

To note, we focused on the effects of altruistic behaviors on individuals’ subjective feelings of warmth in the current study. And we did not found significant similar effects on individuals’ objective physical states (body temperature in Studies 3a and 3b). This also implied that the psychological reactions may be faster and more flexible than physical reactions. However, it is possible that the physical states could get feedbacks from psychological states when the timeline is extended concerning the close interactions of physical and psychological states (e.g., Edwards and Cooper, 1988) and the prospective treatment of biofeedback (e.g., Lagos et al., 2013). In addition, we have mentioned in the introduction as well as in the discussion section that an increase in warmth feelings could be helpful for individuals who were coping with cold environment. Warmth feelings could serve as an easily accessible comfort for them especially in some crisis situations. On the other hand, it could become a problem when individuals optimistically perceived the environment as warm but failed to respond to the important environmental cue about temperature. This indicates that a raise of warmth feelings may actually become a double-edge sword in some cases. And it is interesting and prospective to explore the other edge of the sword in future studies.

In summary, the lingering fragrance effect is an important perspective for understanding the strategy in coping with a threat. The interaction between physical and psychological systems makes it possible to change the physical state through the mobilization of internal psychological resources. Under the crisis circumstances with limited conditions, using a psychological resource to resist the bad impacts of a crisis could be a more reasonable or even a single possible strategy to cope with threats. As the results showed, active altruistic behavior is a significant way for people to resist the cold environment. According to the Harry Truman effect, a person’s potential can be activated under certain circumstance (Seligman, 2002). Equally, a person can activate his/her own internal psychological potential to cope with the intense threat and pursue positive results.

### Future Directions

The exploration of the proposed immediate self-reward model of altruistic behaviors could be expanded from two aspects. First, the crisis situations and the experimental settings in the current research were all related to coldness. In some cases, coldness could even be a significant threat to survival (e.g., Hurricane Sandy in Study 1 and the earthquake in Studies 2a and 2b). In such cases, increased feelings of ambient warmth were regarded as a reward for the individuals. The specific reward and the corresponding psychological processes might change with different adverse situations. For example, coolness could become a more comfortable state when the environment was extremely hot. Future research could help to explore different contents of the lingering fragrance effect and to offer more evidence for an immediate internal reward system.

Second, further studies could be conducted on the rewarding nature of altruism. For example, a positive cycle might be established in which increased warmth perception following altruistic behaviors result in future altruistic behaviors. Moreover, de Quervain et al. (2004) found that effective altruistic punishment was related to stronger activation of the dorsal striatum. Similarly, neuroscientific studies would be helpful in finding out whether performing an altruistic behavior would activate the same region or some other reward-related brain regions.

## Ethics Statement

The research project was approved by the Ethics Committee of Department of Psychology, Peking University. Studies 3a, 3b, and 4 involved helping disadvantaged groups (i.e., children from low-income migrant workers’ families, students from a remote and poor town, and children with leukemia). All of the helping scenarios used in the studies (including refining education materials, sending postcards, and donations) were actually carried out.

## Author Contributions

T-YH, JL, HJ, and XX should be considered as joint first authors as they contributed equally to the research.

## Conflict of Interest Statement

The authors declare that the research was conducted in the absence of any commercial or financial relationships that could be construed as a potential conflict of interest.

## Footnotes

1 http://www.mturk.com

2 Participants in Study 2b took the experiment in group of 4 (three participants and one confederate) and they needed to act to share food during the experiment. Although participants were asked not to interact with others, it was possible that their reactions were influenced by other participants. To rule out this effect, we run an HLM test with a contextual model. The average number of packs within each group was regarded as the contextual variable. Results showed that the effects of altruism manipulation was marginally significant, *t*(77) = 1.84, *p* = 0.069. The effect of contextual variable did not reached significance, *t*(77) = 0.18, *p* = 0.860. The results remained when negative emotions, perceived severity and uncertainty of the scenario were included in the model.
